# A Comparative Study on Survivors Versus Non-Survivors Among Diabetic Patients Having Mucormycosis

**DOI:** 10.7759/cureus.47932

**Published:** 2023-10-29

**Authors:** Anbumathi S, Karthikeyan Govindarajan, Yogesh S, Pranav Lokesh G Parivallal, Sathyanarayana Hariharan, Atif Khaleel, Praveen T SB, Katyayani Goswami, Pushpa Saravanan, Dharmarajan Panneerselvam

**Affiliations:** 1 Medicine, Madras Medical College, Chennai, IND; 2 Diabetes and Endocrinology, Madras Medical College, Chennai, IND

**Keywords:** mucormycosis, covid-19, angioinvasive disease, diabetes mellitus, inhalation

## Abstract

Introduction

Mucormycosis is a rare opportunistic fungal infection caused by fungi belonging to the Mucorales order and Mucoraceae family. It ranks as the third most prevalent angioinvasive fungal infection, following aspergillosis and candidiasis. This severe infection typically affects individuals with compromised immune systems, including those with hematological malignancies like leukemia and lymphoma, individuals who have undergone stem cell transplants, and people with diabetes mellitus. Individuals in good health are rarely affected, making immunocompromised individuals particularly vulnerable to this potentially fatal fungal disease. The aim of this study was to perform a comparative analysis of survivors versus non-survivors among diabetes patients admitted with mucormycosis.

Methodology

This was a descriptive observational study. A total of 338 patients were enrolled in the study. The study variables included demographics, COVID-19 infection, diabetes mellitus history, steroid use, use of oxygen support, and steam inhalation.

Results

Of the total 338 patients enrolled in the study, 253 (74.9%) were male and 85 (25.01%) were female. The number of survivors were 305 (90.2%) and non-survivors were 33(9.8%). The mean age of survivors was 52.50 ± 11.31 and non-survivors was 54.06 ± 8.54 years. Patients who underwent steam inhalation had a higher chance of survival compared to those who did not undergo steam inhalation and this association was statistically significant (p=0.01). Males showed a higher chance of survival (93.7%) as compared to females (80.0%). The associations between oxygen support, steroid use, and COVID-19 infection with the survival status were statistically non-significant.

Conclusion

There was a strong association between the history of steam inhalation and the outcome of mucormycosis among diabetes patients admitted with mucormycosis. Female patients demonstrated a higher fatality rate than males indicating a significant gender disparity observed in cases. Our findings may help to better identify and treat patients who are at higher risk for severe forms of mucormycosis.

## Introduction

Mucormycosis, a rare yet formidable fungal infection caused by mucormycetes molds, has gained increasing attention due to its severity and association with various risk factors. This paper tells the critical relationship between diabetes and mucormycosis, focusing on the high mortality rates associated with this infection, particularly in diabetic individuals. As we navigate the complexities of this fungal disease, it becomes evident that understanding the factors distinguishing survivors from non-survivors among diabetic patients is paramount for improving patient outcomes.

Mucormycosis can affect virtually any part of the body, with the sinuses, lungs, and brain being common sites of infection [1]. People with diabetes face an elevated risk due to the disease's propensity to weaken the immune system, making it challenging to combat the infection effectively. Other risk factors, such as immunosuppressive drugs, weakened immune systems from various medical conditions, organ transplants, severe burns or trauma, and intravenous drug use, further compound the susceptibility to mucormycosis [2]. The connection between diabetes and mucormycosis is particularly concerning, as up to 70% of individuals with mucormycosis also have diabetes [3]. High blood sugar levels not only weaken the immune system but also create an environment conducive to mucormycetes' growth [4]. Moreover, diabetic patients often present with comorbidities that increase the risk of mucormycosis, including diabetic ketoacidosis and chronic kidney disease.

Mucormycosis poses varying levels of severity depending on the affected body part, with infections in diabetic individuals often proving more severe and challenging to treat due to the vascular damage caused by high blood sugar levels [5,6]. Antifungal medications constitute the mainstay of treatment, occasionally necessitating surgical intervention to remove infected tissue. Prevention is equally vital in managing mucormycosis, emphasizing the importance of controlling diabetes and minimizing exposure to mold spores [7]. Timely diagnosis and treatment are crucial, as mucormycosis can progress rapidly, especially in diabetic patients with compromised immune systems.

The high mortality rates associated with mucormycosis, especially in diabetic individuals, raise alarms within the medical community. While the overall mortality rate stands at approximately 40-50%, diabetic patients face a staggering 70-80% mortality rate [8,9], primarily attributed to delayed diagnosis, rapid disease progression, and limited treatment efficacy. To address the existing gaps in the literature, our study aims to conduct a comprehensive comparative analysis between survivors and non-survivors among diabetic patients with mucormycosis. While previous research has predominantly described clinical features and outcomes, few studies have directly compared these two groups. Our investigation seeks to fill this void by identifying specific demographic factors, clinical characteristics, or treatment strategies associated with improved survival.

## Materials and methods

Study design and setting

This observational study was actively conducted at Rajiv Gandhi Government General Hospital, Chennai, India, following ethical approval from the Institutional Review Board. The research spanned from April to June 2021 allowing for a comprehensive exploration of mucormycosis cases within this period. This specific timeframe was chosen to capture the mucormycosis cases during the peak of the COVID-19 pandemic, allowing for a focused investigation into the interplay of mucormycosis, diabetes, and COVID-19. The choice of this hospital and timeframe ensured a robust dataset for our investigation.

Study participants and selection criteria

Our study has included all the patients who presented with meeting certain inclusion and exclusion criteria. Patients with established diabetes mellitus and a confirmed diagnosis of mucormycosis via potassium hydroxide (KOH) mount examination were considered confirmed study subjects. To ensure the rigor and relevance of our study, a strong set of selection criteria was employed for the inclusion of participants. These criteria were carefully designed to identify patients who met the specific characteristics and conditions necessary for our investigation while minimizing potential confounding factors. The following criteria were applied to select study participants.

Confirmed Diagnosis of Mucormycosis

Only patients with a confirmed diagnosis of mucormycosis, established through KOH mount examination, were considered for inclusion. These criteria ensured that the study focused exclusively on individuals with documented mucormycosis infections.

Diabetes Mellitus

Patients with a documented history of diabetes mellitus were eligible for inclusion. Diabetes is a known risk factor for mucormycosis, and its presence was a key criterion for establishing a relevant patient population.

Exclusion of Immunocompromised Conditions

Individuals with known immunocompromised conditions, such as HIV/AIDS or chemotherapy-induced immunosuppression, were excluded from the study. This exclusion was necessary to isolate the impact of diabetes and other factors on mucormycosis risk without the interference of additional immunological variables.

Exclusion of Other Confounding Factors

Patients with conditions or factors that could potentially confound the analysis, such as organ transplantation or long-term corticosteroid use, were excluded from the study to maintain the clarity and specificity of the research focus. These criteria ensured that the study population was representative of the research objectives and minimized the potential for bias in the findings. Exclusions were made to account for immunocompromised conditions, minimizing external variables that could influence the study's outcomes.

Data sources and measurement of variables

In our quest to provide a comprehensive understanding of the complex landscape of mucormycosis, we diligently gathered data from 338 eligible patients, meticulously documenting the information using Microsoft Excel (Microsoft® Corp., Redmond, WA). Key variables, such as age, gender, recent COVID-19 infection history, documented diabetes, steroid use during COVID-19 treatment, oxygen support requirements, and the practice of steam inhalation within a month before admission, were systematically recorded. By sourcing this data, we aimed to provide a comprehensive overview of mucormycosis cases in relation to these critical variables, facilitating a refined analysis.

This methodology can be delineated as follows, data collection and categorization, encompassing a multitude of demographic and clinical variables. These variables, including age, gender, COVID-19 status, steroid use, oxygen support, steam inhalation practices, comorbidities, infection site, and clinical symptoms, were thoughtfully categorized, rendering the extensive dataset manageable. For instance, age groups were carefully defined, and gender was distinctly partitioned into "male" and "female." Likewise, the COVID-19 status was systematically categorized as "positive" or "negative" through a proper history-taking process. Our dedication to detail extended to the application of the KOH method, a critical facet of the study, which was used to determine the presence or absence of mucormycosis. By employing KOH to process patient samples, this method selectively dissolved non-fungal components while preserving fungal structures, notably hyphae. The presence of these fungal elements under microscopic examination indicated a positive diagnosis of mucormycosis, whereas their absence denoted a negative result.

Demographic variables, which included age and gender, not only provided valuable insights into the patient population's distribution across different age groups and gender ratios but also acted as crucial determinants for subsequent analyses. Moreover, clinical history and treatment variables, encompassing COVID-19 status, steroid use, oxygen support, and steam inhalation practices, collectively contributed to a comprehensive understanding of the patient's experiences and the intricate landscape of treatment interventions, particularly in the context of mucormycosis and concurrent COVID-19 infections. Our comprehensive data collection also encompassed comorbidity variables, including conditions such as hypertension, asthma, cardiac disease, and others. These variables signified the presence or absence of concurrent health issues within the patient cohort and played a vital role in the holistic assessment of overall health status. Additionally, variables associated with the site of mucormycosis infection were systematically categorized, providing critical insights into the diverse anatomical locations affected by the disease, with data thoughtfully presented as percentages for each specific infection site. The comprehensive array of clinical symptoms captured in our study, ranging from nasal blockage and facial pain to headaches, contributed to a nuanced and sophisticated understanding of the multifaceted clinical presentation of mucormycosis. Our study ultimately shifted its focus to the analysis of survival outcomes among the patients, venturing into the intricate relationships between variables and the ultimate survival status.

Statistical analysis

Our analysis applied Statistical Package for the Social Sciences (IBM SPSS Statistics for Windows, IBM Corp., Version 29.0, Armonk, NY), a robust statistical tool. This rigorous analytical approach ensured that our findings were statistically sound and clinically relevant. The study involved a comprehensive statistical analysis of data collected from mucormycosis patients. The initial phase focused on describing the demographic characteristics, comorbidities, infection sites, and clinical symptoms of the participants. For the demographic data, frequencies and percentages were calculated, offering a clear picture of the age and gender distribution of the patient, as well as the prevalence of comorbidities and the anatomical locations of the infections. Subsequently, a logistic regression analysis was conducted to assess the relationship between selected variables and the survival status of the patients.

## Results

The age distribution of the 338 patients included in the study varied significantly. Most patients fell within the age range of 41-60 years, with 97 patients (28.7%) aged between 41 and 50, and 105 patients (31.1%) falling within the 51-60 age group as shown in Table 1. Patients aged 31-40 also constituted a substantial portion, with 45 individuals (13.3%), while those in the 61-70 age bracket accounted for 69 patients (20.4%). A smaller number of patients were distributed across other age groups, including 21-30 years (seven patients, 2.1%), 71-80 years (14 patients, 4.1%), and less than 20 years (one patient, 0.3%). Among the patients studied, gender distribution showed a clear predominance of males, with 253 male patients (74.9%) compared to 85 female patients (25.1%).

**Table 1:** Demographic distribution of study subject

Demographic	Frequency(n)	Percentage (%)
			Age Distribution		
<20	1	0.3%
21-30	7	2.1%
31-40	45	13.3%
41-50	97	28.7%
51-60	105	31.1%
61-70	69	20.4%
71-80	14	4.10%
Gender Distribution		
Female	85	25.10%
Male	253	74.90%

In this study, the treatment and clinical history of mucormycosis patients in relation to their COVID-19 status, steroid use, oxygen support, and steam inhalation practices were examined. Most of the patients, representing 78.1% as shown in Table 2, had a positive history of COVID-19 infection, underscoring the relationship between these two health challenges. Approximately half of the patients, accounting for 49.4%, reported the use of steroids during their treatment for COVID-19, shedding light on the prevalence of steroid usage in this patient population. Oxygen support was required by 44.1% of the patients during their COVID-19 treatment, indicating the severity of respiratory involvement. Steam inhalation emerged as a common practice among the study participants, with 59.5% reporting its use within one month before admission. These results provide valuable insights into the treatment and clinical background of mucormycosis patients, offering a comprehensive perspective on their COVID-19 experiences and related interventions, which are pivotal factors to consider when assessing the outcomes and associations in this study.

**Table 2:** Treatment and clinical history in mucormycosis patients during COVID-19

Treatment and clinical history		Frequency (n)	Percentage (%)
COVID-19 infection	Negative	74	21.9%
	Positive	264	78.1%
Steroid use	No	171	50.6%
	Yes	167	49.4%
Oxygen support	No	189	55.9%
	Yes	149	44.1%
Steam Inhalation	No	136	40.5%
	Yes	200	59.5%

Table 3 explored the comorbidities prevalent among the mucormycosis patients, revealing a spectrum of underlying health conditions. Hypertension was the most common comorbidity, affecting 61 patients (18%). Asthma was observed in 12 patients (3.5%), while cardiac disease was present in 17 patients (5%). Acute kidney injury (AKI), thyroid disorders, renal stones, and chronic kidney disease (CKD) were less common, each affecting only two patients (0.3%). Notably, a substantial proportion of patients, accounting for 217 individuals (64.2%), did not present any of the listed comorbidities.

**Table 3:** Comorbidities of study participants

Comorbidities	Frequency (n)	Percentage (%)
Hypertension	61	18%
Asthma	12	3.5%
Cardiac disease	17	5%
AKI (acute kidney injury)	2	0.5%
Thyroid dysfunction	8	2.3%
Renal calculi	2	0.5%
CKD (chronic kidney disease)	2	0.5%
No comorbidities	217	64.2%

The infection distribution of mucormycosis infections within the study population exhibited a diverse pattern. The most affected site was the rhino-orbital region, accounting for 158 cases (47%), followed by rhino-sinusal involvement in 71 cases (21%). Rhino-orbito-cerebral presentation was observed in 51 cases (15%) as seen in Table 4, indicating a significant proportion of cases with potentially severe central nervous system involvement. Pulmonary manifestations were documented in 34 cases (10%), highlighting the respiratory implications of the infection. The oral cavity was affected in 20 cases (6%), while cutaneous involvement was seen in seven cases (2%). These findings underscore the heterogeneous anatomical distribution of mucormycosis.

**Table 4:** Distribution of Mucormycosis involvement in different infection sites

Infection sites	Frequency (n)	Percentage (%)
Rhino-Orbital Region	158	47%
Rhino-Sinusal	71	21%
Rhino-Orbito-Cerebral	51	15%
Pulmonary	34	10%
Oral	20	6%
Cutaneous	7	2%

The clinical presentation of mucormycosis patients encompassed a spectrum of symptoms showing the nature of the disease. The most reported symptom was nasal blockage or congestion, affecting 60% of patients (203 cases). Facial pain was also prevalent, with 64% (216 cases) of patients experiencing this discomfort. Headache was another prominent symptom, reported by 69% of patients (233 cases). A foul smell was noted in 28% of cases (95), while orbital pain was observed in 44% (149 cases). Numbness was documented in 34% of patients (115 cases), and redness around the eyes, nose, and sinus tract on the face was seen in 12% of cases (41). Paresthesia, blurred or double vision, toothache, and swollen, infected gums were relatively less common but still present, affecting 8% (27 cases), 4% (14 cases), 6% (20 cases), and 6% (20 cases) of patients, respectively. Discoloration of the skin was observed in 2% of cases (7), while respiratory symptoms such as cough (24%, 81 cases), shortness of breath (8%, 27 cases), and hemoptysis (6%, 20 cases) were also documented. These diverse clinical symptoms highlight the varied and often complex clinical presentation of mucormycosis. The detail of the same has been depicted in tabular form in Table 5.

**Table 5:** Clinical symptoms of Mucormycosis

Clinical symptoms	Frequency (n)	Percentage (%)
Nasal blockage or congestion	203	60%
foul smell	95	28%
Facial Pain	216	64%
Numbness	115	34%
Redness around eyes nose, sinus tract on face	41	12%
Orbital pain	149	44%
Headache	233	69%
Paresthesia	27	8%
Blurred or double vision	14	4%
Toothache	20	6%
Swollen, infected gums	20	6%
Discoloration of skin	7	2%
Cough	81	24%
Shortness of breath	27	8%
Hemoptysis	20	6%

Again, several factors were examined in relation to the survival outcomes of mucormycosis patients as shown in Table 6 to find the association between selected variables and the survival status of study participants. Notably, the practice of steam inhalation emerged as a significant factor associated with improved survival. Patients who underwent steam inhalation exhibited a notably higher chance of survival, as evidenced by an odds ratio of 2.701 (95% CI: 1.273, 5.731). This statistic implies that individuals who did not undergo steam inhalation had approximately 2.7 times higher odds of not surviving, with this association being statistically significant (p-value = 0.01). Gender also played a pivotal role in survival outcomes, with male participants demonstrating a higher likelihood of survival (93.7%) compared to their female counterparts (80.0%). The odds ratio of 3.703 (95% CI: 1.777, 7.715) underscored those females had approximately 3.7 times higher odds of not surviving compared to males, with this association highly significant (p-value < 0.001).

**Table 6:** Strength of association of selected variables and the survival status of study participants

Variables		Survival status		Odds ratio	p-value
		Number (%)		(95% Confidence Interval)	
		Survivors	Non-survivors		
Steam inhalation	Yes	188 (94.0)	12 (6.0)	1	0.01
	No	116 (85.3)	20 (14.7)	2.701 (1.273, 5.731)	
						Gender	Male	237 (93.7)	16 (6.3)	1	<0.001
Female	68 (80)	17 (20)	3.703 (1.777, 7.715)								
				Oxygen support	Yes		131 (87.9)	18 (12.1)	1.594 (0.774, 3.280)	0.206	
No	174 (92.1)	15 (7.9)	1							
					Steroid use	Yes	148 (88.6)	19 (11.4)	1.44 (0.697, 2.975)		0.325
No	157 (91.8)	14 (8.2)	1								
				COVID-19 infection		Yes	237	27	1.291 (0.512, 3.255)	0.588	
-89.8	-10.2										
No	68	6	1							
	-91.9	-8.1								

Conversely, the provision of oxygen support, steroid use, and the presence of COVID-19 infection did not exhibit statistically significant associations with survival status. The odds ratios for oxygen support (1.594), steroid use (1.44), and COVID-19 infection (1.291) suggested that these factors did not significantly impact survival outcomes, as reflected by their respective p-values (0.325 for both oxygen support and steroid use and 0.588 for COVID-19 infection).

These findings provide valuable insights into the factors influencing survival among mucormycosis patients and underscore the potential benefits of steam inhalation and gender-specific considerations in clinical management.

## Discussion

The age distribution observed in our study of diabetic patients with mucormycosis tells that the patients fell within the 41-60 age range, with the age group of 51-60 being the most heavily affected. Conversely, patients aged 60 and above represented a smaller proportion of the study population, accounting for 20.4%. The mean age of survivors and non-survivors did not exhibit a substantial difference. Both groups displayed similar average ages. This finding suggests that age may not be a critical determinant of survivorship in mucormycosis cases, but these findings raise questions about the potential influence of external factors, particularly the COVID-19 vaccination rollout in India [10], on the age distribution of mucormycosis cases. One possible explanation for the lower percentage of patients aged 60 and above is the prioritization of this age group for COVID-19 vaccination in India. As older individuals were considered more vulnerable to severe COVID-19 outcomes, it's possible that a significant portion of them received vaccinations early in the rollout. This proactive vaccination strategy aligns with global public health efforts to protect the elderly from severe COVID-19 consequences. similarly, the higher prevalence of patients in the 41-50 and 51-60 age groups may be due to several factors. Firstly, individuals in these age ranges are not prioritized for COVID-19 vaccination during the initial phases, potentially leaving them more susceptible to mucormycosis as a secondary infection. Secondly, the association between age and diabetes risk is well-established, with the prevalence of diabetes increasing with age. Since our study focuses on diabetic patients, it's conceivable that a higher proportion of individuals in the 41-60 age group had pre-existing diabetes, putting them at greater risk of mucormycosis. This is evident from studies that have explored the impact of COVID-19 vaccination on reducing infection rates in older populations, such as the research conducted by Eyal N et al. [11] and Sezen YI et al. [12]. Regarding gender disparities, our study identified a significant difference in the distribution of mucormycosis cases compared to the research conducted by Rahul Kulkarni et al. [13]. In our study, male patients were more affected by the infection than their female counterparts. Interestingly, despite a lower probability of contracting the disease, female patients exhibited a higher fatality rate than male patients this trend is potentially influenced by biological, behavioral, and occupational factors.

In our observation we can also see that corticosteroids were commonly administered in managing mucormycosis aligns with the findings reported by Sen M et al. [14] and Sharma et al. [15]. Furthermore, in our study, we found that 49.4% of the patients had a history of corticosteroid use during their treatment for COVID-19 infection. This finding aligns with other studies that have reported the use of corticosteroids in the management of COVID-19 and its complications. Corticosteroids are known to have anti-inflammatory properties and can help elevate the severe inflammatory response often seen in COVID-19 patients [16]. Corticosteroid use is a common practice in the management of various medical conditions, including COVID-19. However, their immunosuppressive effects can potentially increase the risk of mucormycosis, particularly in diabetic patients. The decision to use corticosteroids should be made carefully, considering the patient's overall health and the potential risks and benefits. Interestingly, in our study, corticosteroid use did not significantly influence patient survival outcomes. The odds ratio of 1.44 (95% CI: 0.697, 2.975) suggested that the impact of corticosteroid use on patient survival was not statistically significant. This finding may indicate that, while corticosteroids can increase the risk of mucormycosis, other factors, such as prompt diagnosis and appropriate antifungal therapy, can offset their negative effects. The distribution of affected infection sites in our study closely resembled the findings reported by Gupta R et al. [17]. The rhino-orbital region was the most involved site followed by the rhino-sinusal region and rhino-orbito-cerebral involvement. Pulmonary, dental, and cutaneous involvement were observed to varying degrees, highlighting different potential routes of fungal invasion and the susceptibility of distinct anatomical regions on the whole there are other studies that tell the major affect site is the rhino-orbital region [18,19]. Significantly, our study demonstrated a notably higher survival rate compared to the research conducted by Hoenigl M [20], and a study done by A GA et al. [21] tells that only 82% was the recovery rate and this suggests that our management approach, which included early diagnosis, timely antifungal therapy, aggressive surgical debridement, and comprehensive multidisciplinary care, may have played a role in improving patient outcomes.

Our study revealed that COVID-19 infection and mucormycosis have a positive correlation, with 78.1% of patients having a history of positive COVID-19 and 27 subjects did not survive. COVID-19's immunosuppressive effects create a favorable environment for opportunistic infections like mucormycosis, particularly in those with compromised immune systems which is very similar to the study done by Rudrabhatla PK et al. [22]. Additionally, respiratory symptoms and the need for oxygen support in COVID-19 patients increase susceptibility to fungal infections [23]. Surprisingly, oxygen support, often indicative of severe cases, did not independently impact mucormycosis outcomes which go against Jean-Pierre Gangneux et al. [24]; his study shows the high prevalence of invasive pulmonary aspergillosis and candidaemia and high mortality associated with proven or probable (pr/pb) COVID-19-associated pulmonary aspergillosis (CAPA) in mechanically ventilated patients with COVID-19, suggesting that multiple factors contribute to patient outcomes. The negative association shows that the COVID-19 status and history of oxygen support in the patient may not be the most crucial factor in predicting whether someone with mucormycosis will survive or not. Other factors, such as the patient's overall health, the promptness of medical treatment, and the specific strain of the fungus involved, may have a more substantial impact on survivorship in cases of mucormycosis. We also found that steam inhalation emerged as a potential factor associated with improved survivability among mucormycosis patients, especially when coupled with COVID-19. Patients who had undergone steam inhalation exhibited a higher chance of survival compared to those who did not receive this treatment. These findings align with research by Pandit et al. [25], suggesting that steam inhalation may have a positive impact on patient outcomes. Steam inhalation, known for its respiratory benefits, offers a simple and accessible intervention that could complement the clinical management of mucormycosis, particularly in cases involving respiratory symptoms. However, its use should be integrated into a comprehensive treatment plan under the guidance of healthcare professionals.

A significant limitation of this study is the potential for selection bias due to the exclusion criteria, which excluded individuals with hematological illnesses, other immunocompromised conditions, and new-onset diabetes mellitus, which is a serious drawback of this study. This exclusion criterion may create a bias that limits the study's findings to a narrower diabetic group, perhaps missing substantial differences in mucormycosis outcomes among a broader spectrum of diabetes individuals, and also the study's lack of long-term follow-up prevents our understanding of the long-term impacts and recurrence of mucormycosis among survivors. Furthermore, while the sample size of 338 individuals is large, it may lack the ability to discover more subtle relationships or risk factors, especially for rare outcomes such as mucormycosis. Lastly, the fact that this study was conducted at a single hospital is a limitation because it may limit the generalizability of the results to a larger population of diabetic patients with mucormycosis, potentially removing the full diversity of cases that may arise in different healthcare settings.

## Conclusions

Our comparative study has explained several critical facts of mucormycosis in diabetic patients. Notably, we found that age diversity within our study population did not hold significant influence over survival outcomes, prompting further investigation into gender-based survival disparities. Interestingly, the use of corticosteroids, a common practice in managing COVID-19, did not substantially impact patient survival, highlighting the multifactorial nature of these outcomes. The intricate infection distribution of mucormycosis infections underscores the complexity of its presentation, while our study also found steam inhalation as a potential factor associated with improved survival, especially in conjunction with COVID-19, invites further exploration of this simple yet promising intervention. Overall, our study underscores the pressing need for a comprehensive approach to patient care, encompassing heightened awareness, timely diagnosis, swift treatment, and the integration of innovative strategies. This holistic approach is essential for mitigating the significant mortality rates linked to this severe fungal infection in diabetic individuals.
